# Evaluation of Thermal Damage Impact on Microstructure and Properties of Carburized AISI 9310 Gear Steel Grade by Destructive and Non-Destructive Testing Methods

**DOI:** 10.3390/ma14185276

**Published:** 2021-09-14

**Authors:** Kamil Dychtoń, Andrzej Gradzik, Łukasz Kolek, Krzysztof Raga

**Affiliations:** 1 Department of Materials Science, The Faculty of Mechanical Engineering and Aeronautics, Rzeszow University of Technology, Powstancow Warszawy 12, 35-959 Rzeszow, Poland; andrzej_gradzik@prz.edu.pl (A.G.); kolek@prz.edu.pl (Ł.K.); 2 Research and Development Laboratory for Aerospace Materials, Rzeszow University of Technology, Zwirki i Wigury 4, 35-036 Rzeszow, Poland; 3 Pratt & Whitney Rzeszow, Hetmanska 120, 35-078 Rzeszow, Poland; krzysztof.raga@prattwhitney.com

**Keywords:** low-pressure carburizing, case hardening, grinding burn, hardness, gears, Barkhausen noise, laser heating, AISI 9310

## Abstract

Advanced aircraft gearboxes operate under high mechanical loads. Currently, aircraft gears are manufactured from chromium–nickel–molybdenum steel grades such as AISI 9310 or Pyrowear 53. The major causes of gear failure are wear and fatigue cracking. As the crack initiation occurs predominantly on the component surface, the gears are routinely subjected to surface hardening processes such as low-pressure carburizing and case hardening. The gears are manufactured in a multiple operation process, in which teeth grinding is a crucial step. Selection of improper grinding conditions can lead to local heat concentration and creation of grinding burns, which are small areas where microstructure and properties changes are induced by high temperature generated during grinding. Their presence can lead to significant reduction of gear durability. Therefore destructive and non-destructive (NDT) quality-control methods such as chemical etching or magnetic Barkhausen noise (MBN) measurements are applied to detect the grinding burns. In the area of a grinding burn, effects related to the over-tempering or re-hardening of the carburized case may occur. In this paper, the results of the studies on the characterization of microstructure changes caused by local heating performed to simulate grinding burns are presented. The areas with the over-tempering and re-hardening effects typical for grinding burns were formed by laser surface heating of carburized AISI 9310 steel. Analyses of the microstructure, residual stresses, retained austenite content, and non-destructive testing by the MBN method were performed. The correlation between the MBN value and the properties of the modified surface layer was identified. It was also found that the re-hardened areas had similar characteristics of changes in the Barkhausen noise intensity, despite the significant differences in the width of the overheated zone, which depended on the laser-heating process conditions.

## 1. Introduction

Design of advanced aerospace transmission systems is a great challenge for mechanical and materials engineers and manufacturers. A suitable configuration of gears and shafts is required in order to obtain the optimum performance and high load-carrying capacity as well as long operation time and low fuel consumption [1,2].

Advanced gearboxes have a major application in automotive and aircraft transmission systems. Modern airplanes and helicopters are powered by turbofan, turboprop or turboshaft engines. Each turbine-engine-powered aircraft is equipped with more than one gearbox, including the main gearbox (MGB), accessory gearbox (AGB), angle gearbox (AnGB), reduction gearbox (RGB), and accessory drive train (ADT) [1,3,4].

The gears and shafts are the critical rotating components of the gearbox that determine the capability and reliability of the drive system. Each component of the transmission is designed to operate in the demanding service conditions. The requirements arising from application of gears define in turn the required mechanical properties of the material [5].

Gears and shafts are subjected to cyclic loads, which generate tensile stresses in the surface layer and increase the risk of fatigue damage. Therefore, high wear and fatigue resistance within the surface layer is critical to ensure a long operation time of the gears. These properties are obtained by heat treatment or thermochemical treatment, mainly by induction hardening, carburizing, and nitriding. The through-hardening process is used for gears when medium wear resistance and load-carrying capacity are desired. The case-carburized gears feature higher wear resistance and load-carrying capacity, and are implemented when a high dynamic stress resistance on teeth flanks is required [1,6,7,8].

The required properties of case-carburized gears (surface hardness >700 HV, carburizing case depth 0.3–2.0 mm, and compressive residual stresses of about 300 MPa) must be obtained both for the gear flank and in the tooth root [7,9]. Thus, advanced high-strength steel grades are currently used in gear-manufacturing processes. Steel grades such as AISI 9310 (according to the AMS 6265 standard) and Pyrowear 53 (according to the AMS 6308 standard), are typically selected as suitable materials for production of aerospace gears and shafts [5,10,11]. Manufacturing of gears is a complex and demanding process in terms of keeping the quality level up to the applicable standards and specifications [10,11,12]. The production process consists of several processing operations, which can be divided into the following stages:

1. Rough machining;
2. Teeth cutting;
3. Thermochemical treatment;
4. Teeth grinding;
5. Finish machining;
6. Material coating/plating;
7. Non-destructive testing and dimensional tolerance inspection.

Thermochemical treatment and teeth grinding are operations of particular importance to the quality of the gear, which is later verified by non-destructive and destructive inspection methods [13,14,15,16].

The thermochemical treatment—carburizing—is sequenced as the third stage of the manufacturing process. Carburizing has been widely applied due to its capability to produce a hard surface layer that is much more durable in comparison to the layers obtained by other thermochemical processes. Currently, the carburizing case-hardening process is the main thermochemical process used in the manufacturing of gears for aircraft gearboxes. Carburizing and case hardening includes gas carburizing, austenitizing, quenching, sub-zero treatment, and tempering. The carburizing and post-carburizing treatments increase the surface hardness and wear resistance while maintaining high toughness of the core. The main benefit of carburizing case hardening is significantly better compressive residual stresses distribution at the surface and within the case depth in relation to other thermochemical processes. The low-pressure carburizing (LPC) process has been developed and implemented as an alternative to conventional carburizing. The LPC process has numerous advantages, including the prevention of intergranular oxidation (IGO) in the carburized layer, which has a strong negative impact on fatigue strength of the gear teeth [1,7,9,17].

Grinding of the teeth and their finish machining are the final machining steps, which are performed to achieve the required geometry of the gear and meet the dimensional and tolerance requirements. The gear undergoes the final grinding operation after the completion of carburizing and heat-treatment operations as the desired microstructure morphology and residual stress values are obtained. The gear teeth are ground in multiple passes of the grinding wheel under restricted process parameters, so that only the appropriate amount of material is removed within a single pass. Excessive quenching distortions of teeth leads to excessive material removal in one grinding pass, which in turn may lead to the generation of detrimental grinding burns [6,8,18,19,20].

Grinding requires a very large energy input per unit volume of removed material. Proper selection of the process parameters is thus crucial. A large part of energy is converted into heat that is concentrated in the surface layer of the ground material. The accumulation of heat results in a rapid local increase in temperature. The temperature increase in the grinding zone may cause several types of thermal damage to the material in the near-surface areas of the affected components. Damage of case-carburized steel caused by grinding may involve a significant change of surface integrity, microstructure, and properties. Local tempering and reduction of hardness is possible within the hardened case, as well as re-hardening, causing increase of brittleness and undesirable tensile residual stresses that lead to microcrack initiation and a decrease in the fatigue life. The types of grinding-burn defects are usually classified as: surface oxidation, over-tempering, re-hardening, and cracking. Type and severity of damage depend on input parameters and the interaction of mechanical forces, temperature gradient, and metallurgical properties of the material [6,8,13,18,19,20,21].

Oxidation burn is visually distinguishable as a discoloration of the workpiece caused by a thin layer of oxides forming a blue temper scale on the surface. The discoloration usually occurs without a significant microstructure degradation in the hardened case [7,19].

Over-tempering (thermal softening) occurs as the tempering temperature is locally exceeded during grinding process. Over-tempering causes the local reduction of hardness on the gear surface and an increase in tensile stress in the damaged zone [7,15,22].

Re-hardening burn is caused by a phase transformation taking place as the temperature in the grinding zone exceeds the austenitizing temperature. A thin layer of hard and brittle, untempered martensite is created upon transformation of austenite. Formation of martensite induces volume expansion, along with tensile residual stress [7,8,9,15,21,22].

An inaccurately performed grinding process has a strong negative impact on the properties and performance of gears and shafts, and can lead to their reworking or rejection at the stage of quality control. Therefore, the accurate and continuous inspection of grinding processes and ground gears is essential. Several inspection systems have been developed and implemented to detect grinding burns. The oxidation of the ground surface may be detected visually. Non-destructive chemical inspection is commonly used for detection of grinding burns by etching of the ground surface using a 4% solution of nitric acid in alcohol—“nital”—and subsequent bleaching of the etched surface in 4–6% hydrochloric acid solution. Nital etching reveals the thermal damage characterized by abnormal microstructural phase transformation as different tones of the etched surface colour. Nevertheless, the application of etching involves a potential risk corresponding to the dimensional changes of the gear. The other disadvantage is related to possible initiation of corrosion and hydrogen embrittlement. Moreover, the etching technique is not capable of detecting deviations in residual stress distributions [15,16,23,24].

Several non-destructive testing (NDT) techniques are used in quality control of carburized case-hardened gears, such as Barkhausen noise analysis, micromagnetic multi-parameter microstructure and stress analysis, eddy current signal, and recently digital image processing [4,15,16,20,25,26,27,28]. The magnetic Barkhausen noise (MBN) technique is presently widely and successfully used as an alternative to chemical etching. MBN is generated by irreversible discontinuous Bloch walls movement induced by cyclic magnetization [20,29]. As the ferromagnetic material is subjected to magnetic excitation, the magnetization is not obtained continuously but in discrete steps, allowing the domain walls to interact and overcome barriers in their path. Sudden changes of magnetization yield electromagnetic and acoustic signals that are detected by an electromagnetic coil or an acoustic transducer. The intensity of this signal is further used to characterize the surface integrity, residual stresses, and microstructure of the material in the surface layer [9,14,15,16,17,20,25,29].

It has been shown that MBN, related to magnetic properties of the material, is also sensitive to chemical composition, microstructure morphology, presence of crystalline defects, residual stresses, and hardness [20,25,26]. Nevertheless, the high sensitivity of the method creates a wide potential for its application, including material characterization and grinding-burn detection. Moreover, MBN has some additional advantages in the evaluation of grinding burns, such as a lower bias compared to visual analysis, which is highly affected by human factor [15,16,17,25].

Calibration of MBN quality-control devices is required for their correct operation. The formation of grinding burns is unpredictable. Hence, it is not recommended to develop calibration data on the basis of the analysis of actual burns. It is therefore necessary to create certain thermal effects in the surface layer in a controlled manner. This can be performed by application of high-power laser-beam energy (laser surface heating) or induction heating [18]. In this paper, the thermal effects simulating grinding-burn damage were produced by means of laser-beam surface heating of carburized case-hardened AISI 9310 steel. The MBN technique was applied to characterize the heat-affected zone. An attempt was made to find a correlation between the microstructure and hardness of the hardened case and the Barkhausen noise signal. Determination of this correlation distinguish over-tempered and re-hardened zones using non-destructive technique.

## 2. Materials and Methods

The material under consideration was the AISI 9310 steel grade developed for low-pressure carburizing and used extensively for aircraft gearing. The chemical composition of the material is listed in Table 1.

The samples were cut off a round bar 100 mm in diameter using a water-cooled band saw and ground to a thickness of 10 mm and surface roughness of Ra = 1.85 μm. The samples were carburized in a multi-segment vacuum carburizing process (Table 2) followed by heat treatment (Table 3).

The process of vacuum carburizing and heat treatment was carried out in a MonoTherm HK.10.gr vacuum furnace (ALD Vacuum Technologies GmbH, Hanau, Germany) and a Linde Cryoflex (Linde plc, Dublin, Ireland) cabinet freezer in the Research and Development Laboratory for Aerospace Materials at Rzeszow University of Technology.

**Figure 1:**
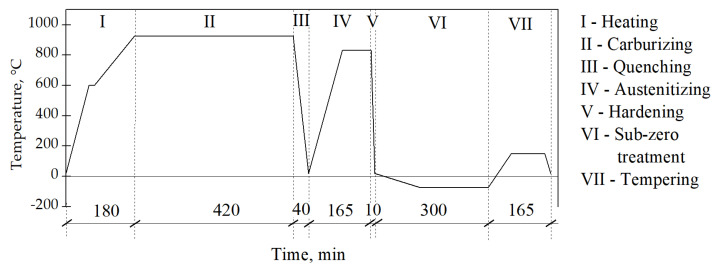
Scheme of the thermo–chemical processing of the AISI 9310 steel.

Prior to the austenitizing process, the sample was electrolytically plated with copper to avoid oxidation. The copper layer was then removed from the sample by grinding preceding the laser-beam processing. The scheme of the thermo-chemical treatment is presented in Figure 1.

**Figure 2:**
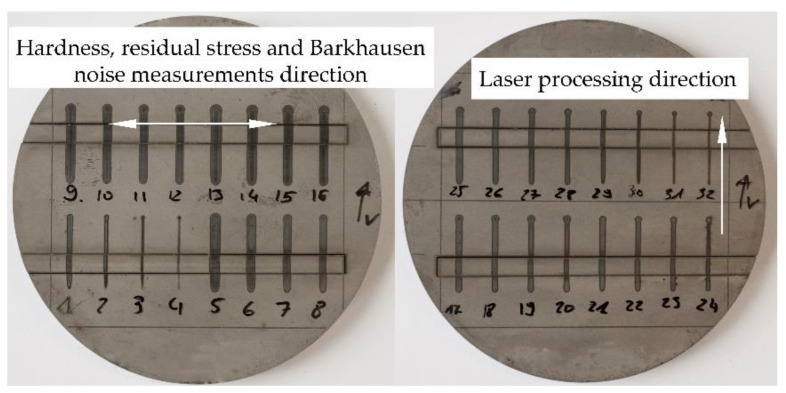
Tracks created by laser surface heating and the direction of surface hardness, residual stresses, and MBN measurements.

Laser surface heating was performed to produce controlled thermal damage on the surface of carburized samples similar to damage generated by grinding burns (Figure 2).

The laser heating of the sample surfaces was performed using an Yb:YAG TRUMPF TruDisk 1000 laser (TRUMPF Laser GmbH + Co., Ditzigen, Germany) with a maximum power of 1 kW and wavelength *λ* = 1030 nm. The diameter of the beam on the surface of the workpiece was 2 mm. Prior to the laser heating, the surface was cleaned with 2-propanol. The processing parameters applied were as follows: laser power *P*—80–320 W, and scanning velocity *v*—250–1250 mm·min^−1^. The power density of the laser beam was in the range of 2.5–7.2 kW·cm^−2^. The process was carried out in an air atmosphere. The local laser irradiation of the workpiece sample generated a series of tracks 30 mm in length (Figure 2) on the surface. For further investigation the tracks exhibiting thermal effects such as oxidation, over-tempering, and re-hardening were selected (Table 4).

Samples subjected to the laser surface processing were analyzed with a Rollscan 350 (Stresstech Ltd., Jyväskylä, Finland) Barkhausen noise analyzer and Microscan and Viewscan software. The measurements were performed using two methods: scanning in the direction transverse to the beam movement during laser processing, and measurement of the root mean square (RMS) in the center of the track. The following conditions for MBN measurements were applied: a magnetizing frequency of 50 Hz at a voltage of 2.5 V for the surface-scanning tests, and a magnetizing frequency of 80 Hz at a voltage of 3.4 V for the center-point tests. During the MBN measurements, the sinusoidal magnetizing current waveform was used.

**Figure 3:**
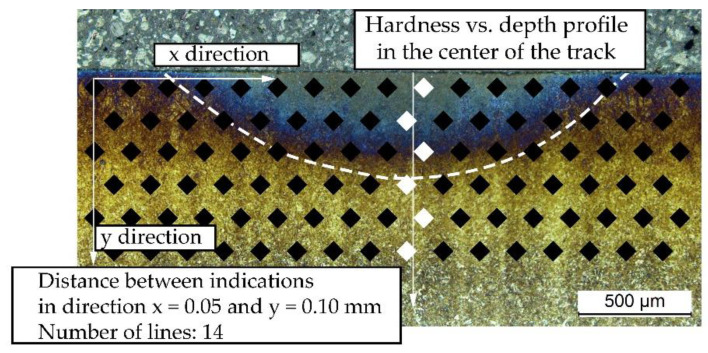
Scheme of the hardness measurement locations on the cross-section of the track.

The hardness measurements of laser-processed samples were performed using an Innovatest Nexus 4303 (Innovatest, Maastricht, The Netherlands) hardness tester. Measurements were carried out with a Vickers indenter at a load of 4.9 N. The measurements of surface hardness were performed across the tracks in the direction transverse to the laser beam’s movement. The distance between hardness measurements was 0.05 mm. Prior to the surface hardness measurements, the samples were polished with a diamond suspension. A hardness map on the cross-section of the tracks also was obtained to determine the hardness distribution in the heat-affected zone. The hardness was measured along the lines parallel to the surface, beginning at a depth of 0.05 mm (Figure 3). The distance between the lines was 0.05 mm, and the distance between the hardness indentation points was 0.1 mm (zig-zag pattern). The samples were prepared by wet grinding with SiC grinding paper and polished with a diamond suspension.

Microstructure examination of samples was performed using a Leica DMI 3000M metallographic microscope (Leica Microsystems, Wetzlar, Germany). Ground and polished samples were subjected to chemical etching with nital reagent (HNO_3_–4 cm^3^ and ethanol 96%–100 cm^3^) to reveal microstructure morphology and the extent of the heat-affected zone created by laser processing.

Residual stress measurements were carried out by the X-ray diffraction method using a PROTO iXRD Combo X-ray diffractometer (Proto Manufacturing Ltd., Oldcastle, Canada) according to the SAE J784a standard. The sin^2^*ψ* method and the *ω* geometry were applied. The following measurement parameters were used: Cr-Kα X-ray lamp; wavelength *λ*Cr_Kα_ = 0.2291 nm; Bragg angle of {211} plane—156.4°; *ψ* angle values—±37.00°, ±31.41°, ±24.00°, ±17.51°, ±13.00°, ±12.00°, ±7.41°, ±6.49°, and 0.00°; aperture diameter—2 mm; exposure time—2 s; number of exposures—40; gain material—*β*-Ti. Residual stresses were determined at the surface of tracks in the direction perpendicular to the laser-processing direction (Figure 2).

The volume fraction of retained austenite at the surface of the samples prior to and after laser processing was also determined by X-ray diffraction. The same radiation source as in the residual stress measurements was used. The aperture diameter was 1 mm. The retained austenite content was determined according to the ASTM E975 standard using {200} and {211} crystallographic planes in martensite, as well as {220} and {200} planes in austenite.

## 3. Results and Discussion

**Figure 4:**
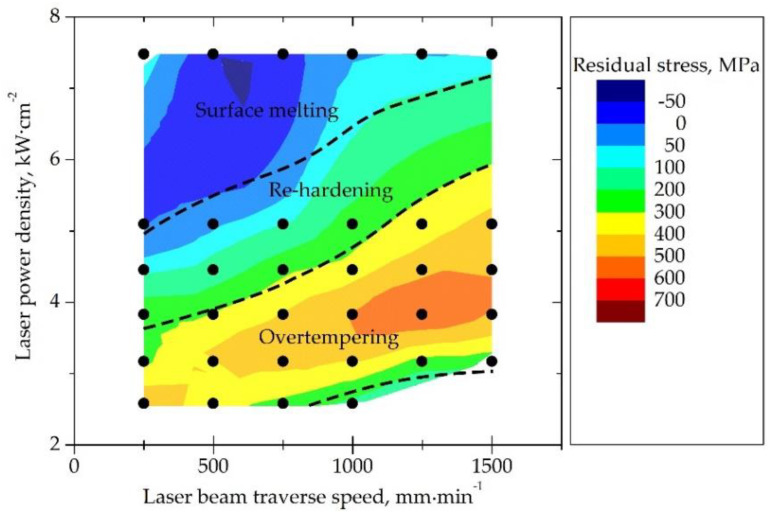
The influence of laser-processing conditions on residual stress values in the irradiated zone of the carburized case-hardened AISI 9310 steel.

Visual analysis of the tracks generated by laser processing revealed the evidence of surface oxidation. The extent and intensity of particular effects such as melting, over-tempering, and re-hardening in the irradiated zone were dependent on the laser-processing conditions of power density and beam traverse speed (Figure 4). Moreover, it was observed that at a constant power density, the width of the track decreased with an increase in the laser beam traverse speed. It was also found that surface melting could occur in the center of the track during laser processing at a power density exceeding 7 kW·cm^−2^ and a beam traverse speed of 250–1500 mm·min^−1^. However, local surface melting did not occur during grinding-burn formation. Hence, no further analysis of such a case was conducted.

### 3.1. Residual Stresses

The residual stresses at the surface of the workpiece prior to laser heating were −62 ± 20 MPa regardless of the measurement direction. It was found that the parameters of the laser-heating process had a significant influence on the value of the residual stresses in the irradiated zone. The value and characteristics of the residual stresses were related to changes in the microstructure: over-tempering or re-hardening effects (Figure 4).

It was noticed that the high tensile residual stresses were typical for the tracks where only over-tempering occurred. An increase in the energy input during laser processing (higher power density and lower traverse speed) induced a re-hardening effect in the track. The tracks that exhibited a re-hardening effect showed tensile residual stresses from 100 to 200 MPa. Relaxation of residual stresses in the range of −50–0 MPa was observed in the area where surface melting occurred (Figure 4).

### 3.2. Microstructure and Hardness

**Figure 5:**
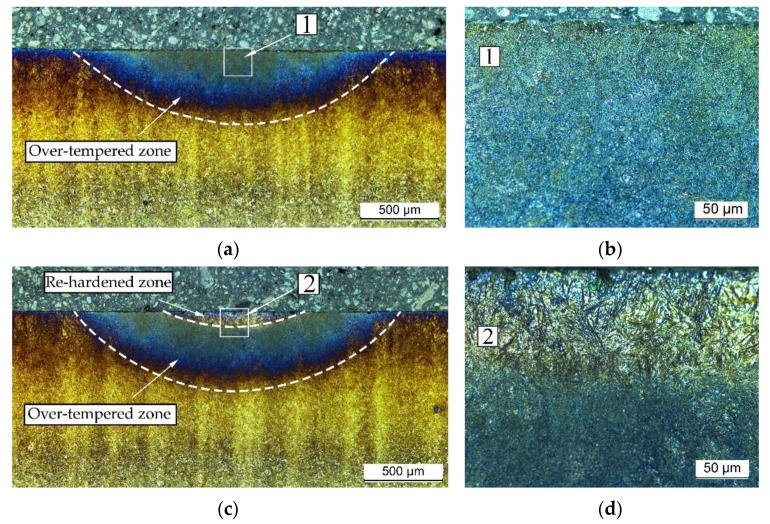

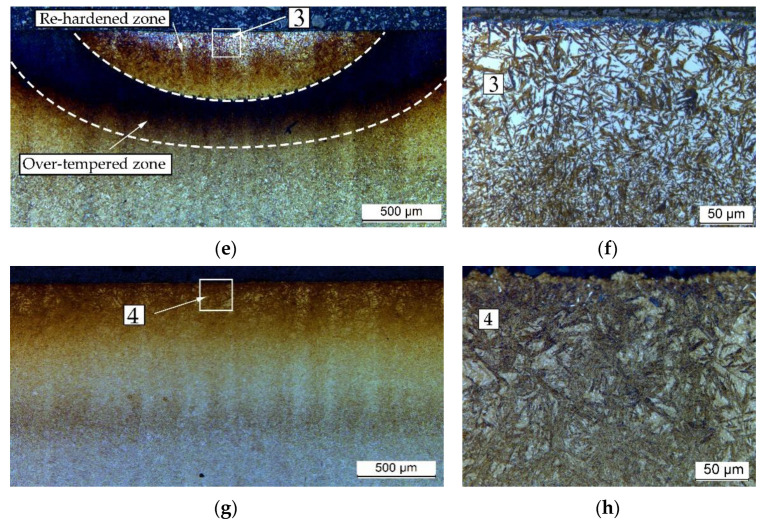
Microstructure of cross-section of tracks created by laser surface heating of the AISI 9310 carburized case-hardened steel: (**a**,**b**) track 1; (**c**,**d**) track 2; (**e**,**f**) track 3; compared to the carburized case (**g**,**h**) prior to the laser heating.

The laser-beam surface heating produced different thermal effects in the carburized case-hardened AISI 9310 steel depending on the process parameters. Microstructure analysis was performed on the tracks, with thermal effects similar to those caused by grinding burns: over-tempering and re-hardening (Figure 5a–f) compared to the microstructure of the carburized case prior to the laser heating (Figure 5g,h). Tracks selected for metallographic and non-destructive investigation by MBN as well as hardness measurements were processed using the parameters presented in Table 4. Microstructure examination and results of the hardness measurements showed that the absorbed laser-beam energy and the associated temperature rise in the surface layer of the sample created a heat-affected zone (HAZ) similar to that formed during welding [20].

**Figure 6:**
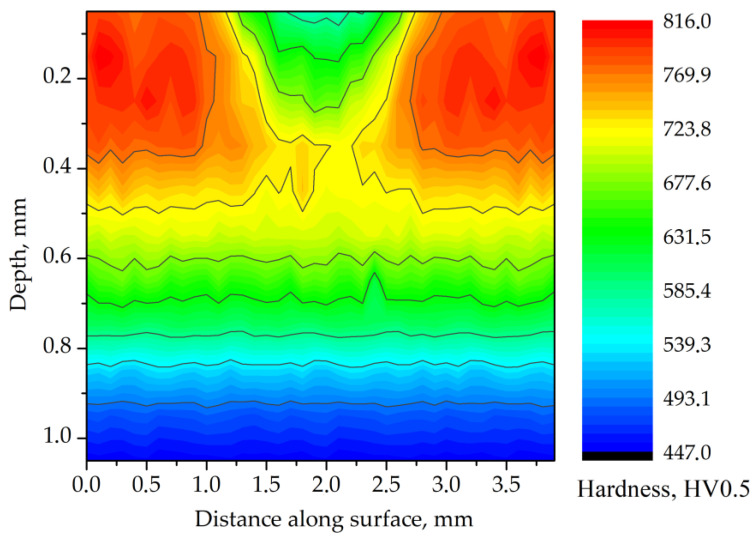
Hardness distribution map for the cross-section of track 1. Processing parameters: laser beam power of 80 W; traverse speed of 250 mm·min^−1^.

Degradation of microstructure in the carburized case had a significant influence on the hardness. The surface hardness of track 1 (Table 4) decreased continuously from 825 HV in the unaffected zone to 525 HV in the center of the track (Figure 6). Significant reduction of hardness indicated local tempering in the laser-irradiated area. The width of the track (HAZ) on the surface was determined to be nearly 2 mm.

**Figure 7:**
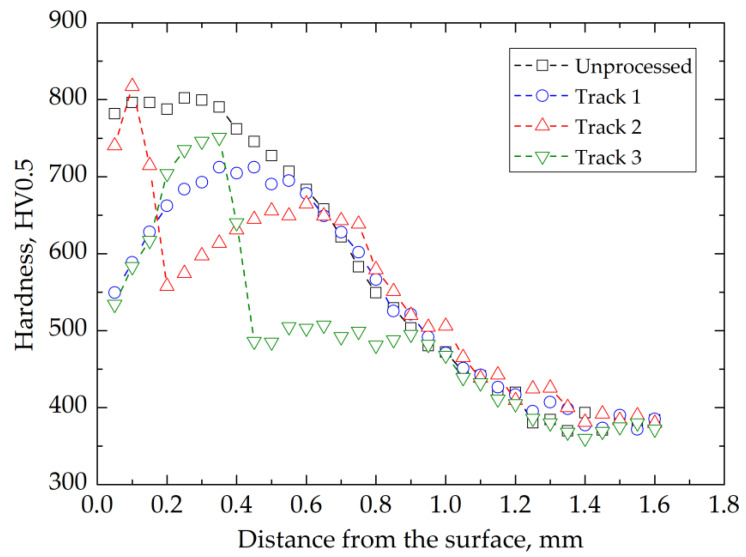
Hardness distribution of the carburized AISI 9310 steel in the tracks created by laser heating as a function of distance from the surface.

A hardness vs. depth profile (Figure 7) was obtained in the center of the laser-processed track. A decrease in hardness was observed up to the 500 µm depth, as the tempered martensite microstructure developed (Figure 5a,b). Examination of the microstructure showed the presence of undissolved carbides near the surface and a lack of retained austenite compared to the unprocessed carburized case, which consisted of tempered martensite, retained austenite, and carbides [1,5,6,7,30,31,32]. The quantitative XRD phase analysis confirmed a low amount of retained austenite in the carburized case (Table 5). The decrease in volume fraction of retained austenite was from 7.8% in the unaffected zone to 3.5% in the center of the track. We therefore concluded that the temperature during laser processing was below Ac_1_. Thus, track 1 was an example of over-tempering-related thermal damage [7]. We also found that below the depth of 0.5 mm, there was no significant change in hardness due to over-tempering in comparison to the unaffected zone (Figure 5a and Figure 7).

Increasing the power density of the laser beam caused a widening of the HAZ up to 2.5 mm for track 2 and 2.75 mm for track 3 (Table 4). Two characteristic regions were identified in the microstructure of track 2 (Figure 5c,d). The first was located in the center of the track, while the second was surrounding it. Observations revealed a significant amount of retained austenite and minor carbide precipitates in the center of the track. The volume fraction of retained austenite in the center of the track determined by XRD phase analysis was 18.1%. During the laser-heating process, the highest temperature in this area exceeded the Ac_1_ value. Subsequently, as a result of rapid cooling below the martensite-start (M_s_) temperature, the hardening occurred in this region. Nevertheless, the martensite-finish (M_f_) temperature was not reached during cooling due to the high carbon concentration in the austenite.

**Figure 8:**
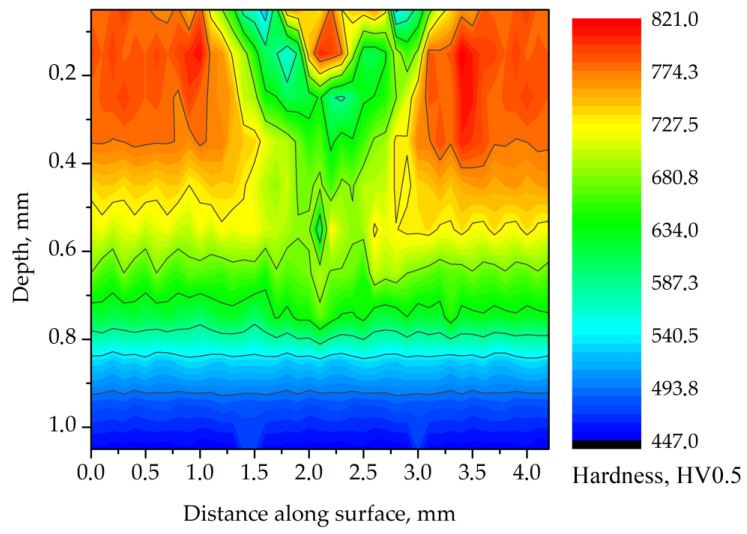
Hardness distribution for the cross-section of track 2. Processing parameters: laser beam power of 140 W; traverse speed of 750 mm·min^−1^.

It was found that the center of the track featured high hardness up to 850 HV (Figure 8). In the zone surrounding the center of the track, the hardness decreased in a continuous manner similarly to track 1, which is typical for over-tempering. The analysis of the hardness vs. depth profile (Figure 7) suggested that the initial decrease of hardness to the depth of 0.1 mm was caused by a high volume fraction of retained austenite. At a depth of 0.3 to 0.6 mm below the surface, the effects of over-tempering and a decrease in hardness of the carburized case were observed. The rapid change of hardness observed between depths of 0.2 to 0.3 mm suggested that this was the region where the over-tempering and re-hardening effects coincided.

**Figure 9:**
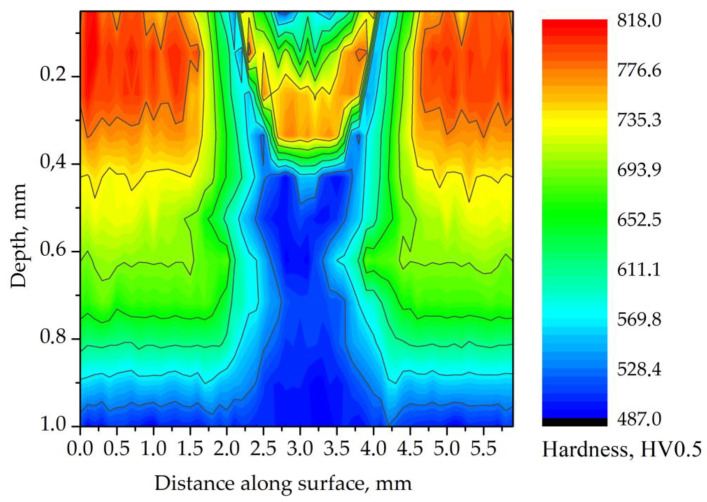
Hardness distribution for the cross-section of track 3. Processing parameters: laser beam power of 160 W, traverse speed of 500 mm·min^−1^.

Track 3 (Table 4) was laser-processed at the highest energy input. Therefore, the highest temperature in the irradiated zone was reached. Microstructure analysis and hardness measurements (Figure 5e,f and Figure 9) suggested that similarly to track 2, two characteristic regions could be distinguished—the central and surrounding zones. In the center of the track, the Ac_1_ temperature was exceeded, resulting in the transformation of tempered martensite to austenite during the laser-heating process and martensitic transformation on cooling. A considerable decrease of the retained austenite volume fraction at the surface, from 57.5% in the center of the track to 18% within 1 mm of it, was a result of uneven temperature distribution in this region during laser processing. Moreover, no carbide precipitations were observed near the surface. This indicated that the maximum temperature in the central zone was significantly higher for track 3 in comparison to track 2.

The microstructure degradation in the heat-affected zone induced non-uniform distribution of hardness in the carburized case of AISI 9310 steel. Surface hardness of the re-hardened zone increased from 525 HV in the center of the track up to 880 HV within a 0.85 mm distance (Figure 9). We therefore concluded that close to the center of the track, the maximum temperature during laser heating significantly exceeded the Ac_1_ value, leading to decomposition of carbides and a local increase in carbon content in the austenitic matrix. Hence, it was not possible to attain the M_f_ temperature, and a large amount of retained austenite remained in the microstructure, causing a significant decrease in hardness [7]. Farther from the center of the track, the Ac_1_ temperature was not exceeded, and thus only effects related to the over-tempering occurred. The hardness increased from 525 HV at the edge of the over-tempered region up to 825 HV in the unaffected carburized case. At the boundary of these two zones, a rapid decrease in hardness from 880 to 525 HV was observed (similarly to track 2).

The analysis of the microstructure and hardness vs. depth profile indicated that the re-hardened zone had a depth of 0.4 mm. In this region, three thermal effects coincided. The first was associated with formation of martensite and retained austenite in the re-hardening process. The second effect corresponded to the gradient of carbon concentration along the depth of carburized case. The third corresponded to the gradient of temperature along the depth. It was therefore problematic to separate the influence of these three effects on a hardness decrease of up to 0.4 mm. At depths of 0.5 to 0.9 mm below the surface, the effects of over-tempering and hardness decrease of the carburized case were observed. The rapid change of hardness observed between depths of 0.4 to 0.5 mm suggested that this was the region where the over-tempering effect coincided with the re-hardening effect.

### 3.3. Magnetic Barkhausen Noise Analysis

**Figure 10:**
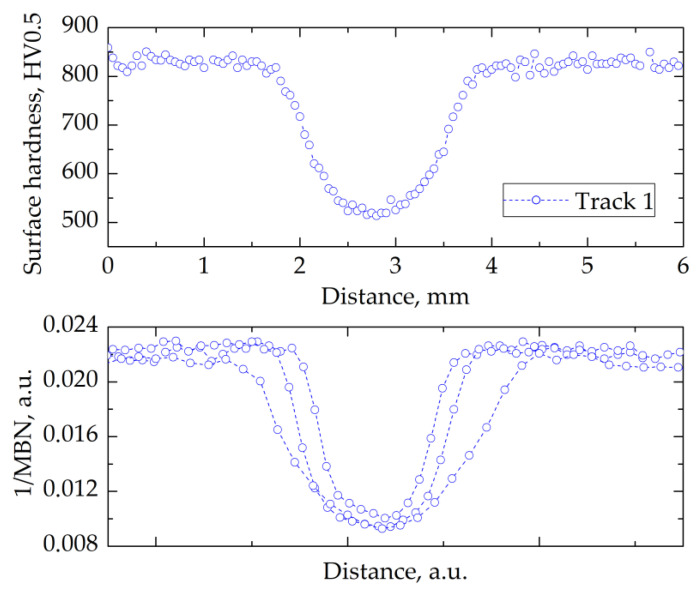
Relation between hardness (upper panel) and normalized 1/MBN (lower panel) as a function of distance (surface coordinate) across the laser-processed track 1.

**Figure 11:**
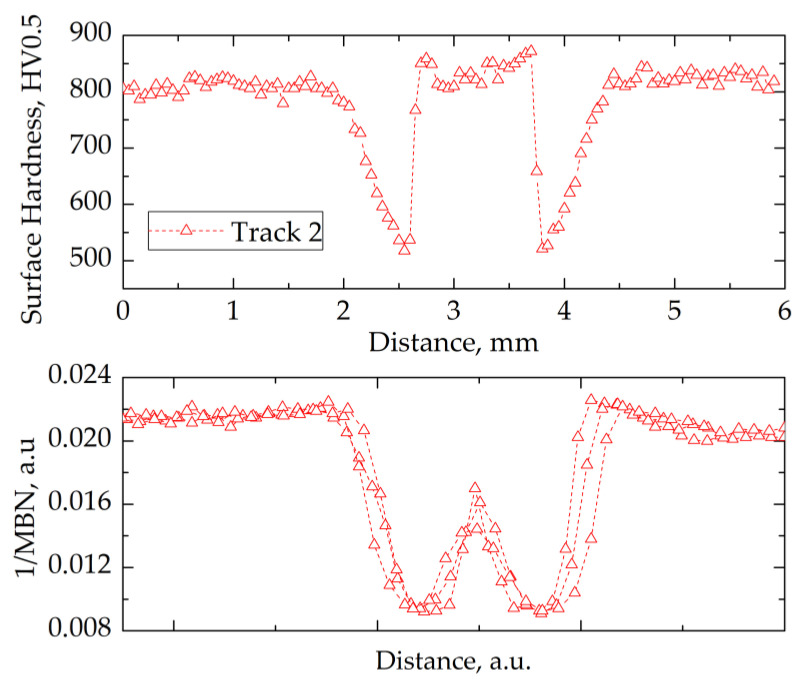
Relation between hardness (upper panel) and normalized 1/MBN (lower panel) as a function of distance (surface coordinate) across the laser-processed track 2.

**Figure 12:**
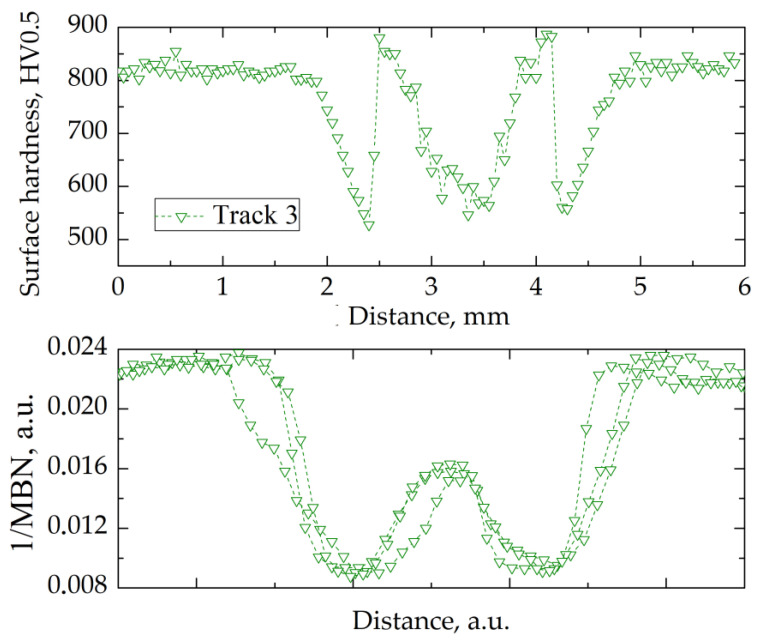
Relation between hardness (upper panel) and normalized 1/MBN (lower panel) as a function of distance (surface coordinate) across the laser-processed track 3.

The results of MBN measurements indicated that the value of the root mean square (RMS) was related to the properties of the sample such as residual stresses and hardness. The MBN value increased with the tensile stresses compared to the stress-relieved material [15,29]. Figure 10, Figure 11 and Figure 12 present the relation between hardness and 1/MBN as a function of distance (surface coordinate) across the track. It was established that for track 1, the 1/MBN values were directly proportional to the hardness (Figure 10). That correlated with the earlier finding that track 1 was affected only by over-tempering.

For tracks 2 and 3 (Figure 11 and Figure 12), a decrease in 1/MBN values was observed in the over-tempered zone, as for track 1. Within the re-hardened zone of the tracks, the 1/MBN value increased due to the high volume fraction of retained austenite. Even though there was a difference in hardness values, tracks 2 and 3 showed a similar dependence of 1/MBN on the measurement distance in the re-hardened zone.

A lack of correlation between the 1/MBN value and surface hardness characterized the transition region between the tempered and re-hardened zones (tracks 2 and 3). The main reason appeared to be the synergistic effect of several factors on the measured Barkhausen noise value. Hardness was measured at equally distant points, while the Barkhausen signal was acquired from a larger area and averaged. Moreover, the residual stresses changed significantly between the over-tempered and re-hardened zones. Hence, it was difficult to find an unambiguous relation between the Barkhausen noise signal value and hardness.

**Figure 13:**
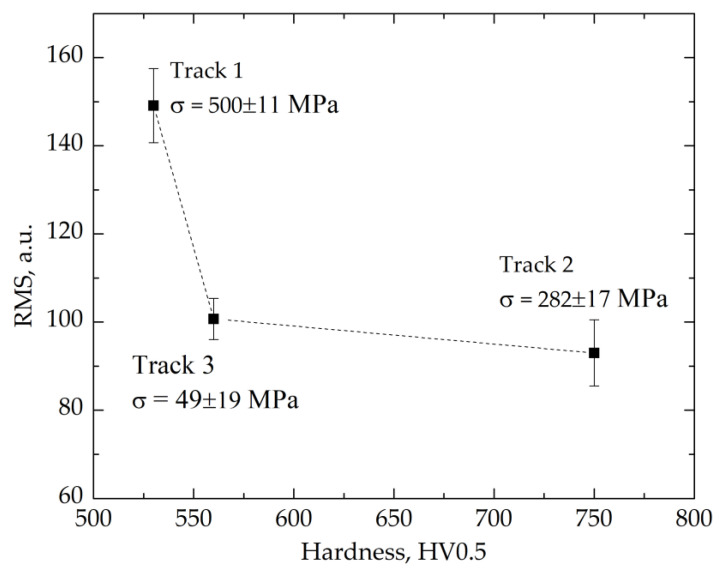
The dependence of the average RMS value, measured in the center of the track, on hardness and residual stresses.

Figure 13 presents the RMS values of the MBN signal as a function of hardness measured in the center points of the track. The MBN signal decreased with increasing hardness, but as the value of residual stresses was not the same, this dependence was not linear. The low BN signal value of track 3 compared to track 1, with an insignificant difference in hardness, was due to the difference in the values of residual stresses. The difference of hardness in the center of tracks 1 and 3 was insignificant. Nevertheless, the RMS value was much lower for track 3. The main reason was the difference in residual stresses between these two tracks, where track 1 was characterized by higher residual tensile stresses formed during over-tempering. On the other hand, tracks 2 and 3 had a similar RMS value despite the hardness difference. Thus, there was a synergy of the impact of residual stresses and hardness on their RMS value [15,29]. The higher content of retained austenite in track 3 (57.5%) compared to track 2 (18.1%) also was likely to affect the RMS value.

As mentioned in [27], specific and reproducible grinding burns are difficult to generate. Therefore, the artificial defects must be generated in controlled manner. The artificial grinding burns generated by laser surface heating in this paper produced changes in the carburized case physically similar to the real defects. The analysis showed that the heat generated by pass of the laser beam could cause the appearance of over-tempered and re-hardened zones in the carburizing layer. Moreover, the laser-beam surface heating enabled easier control of the energy input compared to grinding. Therefore, this technique was chosen to produce artificial grinding burns.

The MBN technique applied in this work allowed to evaluate the microstructure change of carburized case-hardened steel based on the alteration of magnetic properties. Therefore, it is assumed that it can be used for the detection of grinding burns on the surface of gears with similar carburized cases to flat samples used in this research.

## 4. Conclusions

The application of laser surface heating for carburized case-hardened AISI 9310 steel enabled the generation of effects similar to grinding burns in a controlled manner. Changes in the microstructure of the carburized case occurred in the heat-affected zone. The extent of the heat-affected zone was related to the parameters of the laser-heating process. The laser-heating conditions applied in this work allowed us to induce phase transformations typical for over-tempering and re-hardening.

The re-hardened zone was located in the center of the track generated by the laser surface heating. Its range increased with the laser-beam power density. At the same time, the temperature in the center of the heated zone was well above the Ac_1_ value, which led to the dissolution of carbides and an increase in the retained austenite content after cooling.

The over-tempering effect and related phase transformations led to development of tensile stresses between 100 and 600 MPa in the laser-heated zone.

The results of hardness and magnetic Barkhausen noise measurements in the transverse direction to the laser-beam movement suggested the existence of a correlation between the hardness and BN signal intensity. It was found that the inverse of the MBN (1/MBN) value was directly proportional to the hardness value on the surface of the track created in the laser-heating process.

Despite differences in the width of the re-hardened zone, the characteristics of Barkhausen noise changes on the surface of the heat-affected zone remained similar. Thus, it was possible to detect the over-tempered and re-hardened zones with a non-destructive technique, and to distinguish them based on the analysis of the course of the Barkhausen signal change during surface scanning.

The BN measurements in the center of the tracks created by laser surface heating also showed an increase in the Barkhausen signal with decreasing hardness and increasing stress.

## Figures and Tables

**Table 1:** Chemical composition of the studied AISI 9310 steel.

Element, wt %									
C	Ni	Cr	Mo	Cu	Mn	Si	S	P	Fe
0.13	3.18	1.21	0.11	0.07	0.57	0.28	0.003	0.008	Bal.

**Table 2:** Low-pressure carburizing multi-segment process parameters.

Temperature, °C	925
Austenitizing prior to carburizing, min	73
Number of cycles	20
Diffusion segments duration, min	326
Boost segments duration, sec	1260
Total process time, min	420

**Table 3:** Heat-treatment process after low-pressure carburizing.

Heat-Treatment Operation	Temperature, °C	Time, min
Austenitizing	830	60
Quenching in oil	40–50	~10
Sub-zero treatment	−75	180
Tempering	150	60

**Table 4:** Parameters of the laser surface-heating process for the tracks selected for hardness and MBN measurements and microstructure examination.

Track	Laser Power, W	Power Density at the Surface of the Sample, kW·cm^−2^	Laser Beam Traverse Speed, mm·min^−1^
1	80	2.5	250
2	140	4.5	750
3	160	5.1	500

**Table 5:** Volume fraction of retained austenite in unprocessed areas of the carburized AISI 9310 steel in tracks 1 ÷ 3 produced by laser surface heating.

Track	Retained Austenite, %
Unprocessed area	7.8 ± 0.8
1	3.5 ± 0.5
2	18.1 ± 1.3
3	57.5 ± 0.9

## Data Availability

The data presented in this study are available on request from the corresponding author.
